# Systemic Autoimmunity Remodels Microbiota-Reactive CD4⁺ T Cell Immunity

**DOI:** 10.64898/2026.10.17.759871

**Published:** 2026-10-19

**Authors:** Maricielo J. Vaella-Alarcon, Miki Kume, Silvia R. Davila-Paico, Luis F. Soto-Ugaldi, Katherine S. Saldana-Morales, Gillian L. McClennen, Jin Din, Samantha St Jean, Daniel F. Zegarra-Ruiz

## Abstract

The gut microbiota contributes to autoimmune disease, yet how systemic autoimmunity reshapes microbiota-reactive CD4⁺ T cell responses and how these cells influence disease progression remain poorly understood. Previous studies have linked microbiota-reactive T cells and lupus-associated pathobionts to disease pathogenesis, but the pathways that regulate the accumulation, differentiation, and pathogenicity of microbiota-reactive T cells during autoimmunity remain unclear. Here, we show that systemic autoimmunity drives the expansion, accumulation, and functional differentiation of microbiota-reactive CD4⁺ T cells. Using segmented filamentous bacteria tetramers, we identify antigen-defined microbiota-reactive CD4⁺ T cells that undergo systemic accumulation and Th1/Tfh polarization during disease. We further identify reactivation of intestinal dendritic cell trafficking as a mechanism linking systemic autoimmunity to the accumulation of microbiota-reactive CD4⁺ T cells in peripheral tissues. In contrast to commensal-reactive T cells, CD4⁺ T cells conditioned by the lupus-associated pathobiont *Enterococcus gallinarum* markedly exacerbate autoimmunity following adoptive transfer through IL-21- and IFNγ-dependent mechanisms. Together, these findings demonstrate that systemic autoimmunity reshapes microbiota-reactive CD4⁺ T cell immunity and establish microbial context as a critical determinant of microbiota-reactive CD4⁺ T cell pathogenicity.

**SIGNIFICANCE STATEMENT:**

Microbiota-reactive CD4⁺ T cells contribute to host defense and have been implicated in autoimmune diseases, but how systemic autoimmunity alters these responses has remained unclear. We show that systemic autoimmunity promotes the expansion, accumulation, and differentiation of microbiota-reactive CD4⁺ T cells by reactivating intestinal dendritic cell trafficking. We further demonstrate that microbial context determines the pathogenic potential of these cells, with pathobiont-conditioned CD4⁺ T cells acquiring disease-promoting activity. These findings identify a mechanism by which chronic autoimmune inflammation reshapes host-microbiota adaptive immunity and influences disease progression.

